# Autoimmune Encephalitis at the Neurological Intensive Care Unit: Etiologies, Reasons for Admission and Survival

**DOI:** 10.1007/s12028-016-0370-7

**Published:** 2016-12-27

**Authors:** Gayane Harutyunyan, Larissa Hauer, Martin W. Dünser, Anush Karamyan, Tobias Moser, Slaven Pikija, Markus Leitinger, Helmut F. Novak, Eugen Trinka, Johann Sellner

**Affiliations:** 1 Department of Neurology, Christian Doppler Medical Center, Paracelsus Medical University, Salzburg, Austria; 2 Department of Psychiatry, Christian Doppler Medical Center, Paracelsus Medical University, Salzburg, Austria; 3 Department of Critical Care, University College of London Hospital, London, UK; 4 Department of Neurology, Klinikum rechts der Isar, Technische Universität München, Munich, Germany; 5 Department of Neurology, Christian Doppler Medical Center, Paracelsus Medical University, Ignaz-Harrer-Str. 79, 5020 Salzburg, Austria

**Keywords:** Autoimmune encephalitis, Intensive care unit, Mortality, Status epilepticus, Immune-mediated

## Abstract

**Background:**

Early recognition and treatment of autoimmune encephalitis (AE) has become an essential issue in clinical practice. However, little is known about patients with deteriorating conditions and the need for intensive care treatment. Here, we aimed to characterize underlying aetiologies, clinical symptoms, reasons for intensive care admission, and mortality of critically ill patients with AE.

**Methods:**

We conducted a retrospective chart review of all patients with “definite” or “probable” diagnoses of AE treated at our neurological intensive care unit between 2002 and 2015. We collected and analyzed clinical, paraclinical, laboratory findings and assessed the mortality at last follow-up based on patient records.

**Results:**

Twenty-seven patients [median age 55 years (range 25–87), male = 16] were included. Thirteen (48%) had “definite” AE. The most common reasons for admission were status epilepticus (7/27, 26%) and delirium (4/27, 15%). One-year survival was 82%, all five deceased were male, and 3 (60%) of them had “probable” disease. The non-survivors (median follow-up 1 year) were more likely to have underlying cancer and higher need for respiratory support compared to the survivors (*p* < 0.041, and *p* = 0.004, respectively).

**Conclusions:**

Clinical presentations and outcomes in critically ill patients with AE are diverse, and the most common leading cause for intensive care unit admission was status epilepticus. The association of comorbid malignancy and the need for mechanical ventilation with mortality deserves further attention.

**Electronic supplementary material:**

The online version of this article (doi:10.1007/s12028-016-0370-7) contains supplementary material, which is available to authorized users.

## Introduction

Encephalitis is a life-threatening medical condition of various etiologies, which affects patients of all ages and results in substantial morbidity and mortality worldwide [1, 2]. Along with recent advances in diagnostic testing, autoimmune conditions have been receiving increasing recognition as causes of encephalitis [3]. Autoimmune encephalitis (AE) poses a diagnostic challenge because of its heterogeneous clinical presentations that include neurological, psychiatric, and general medical conditions. In addition, at this time antibody testing to confirm AE is only available at specialized centers. Direct consequences are an inconsistent coverage for antibody testing and, in many cases, delayed obtainment of results [4, 5]. Timely diagnosis and treatment is, however, essential for favorable outcome [6–8].

Patients with AE often develop life-threatening complications that necessitate intensive care unit (ICU) admission [9, 10]. Notably, while ICU admission has been shown to be associated with poor outcome, little is known about clinical presentations and radiological and laboratory findings of those patients admitted to the ICU. In this study, we aimed to characterize underlying etiologies and the spectrum of clinical symptoms in critically ill patients with AE, their reasons for ICU admission, and evaluated which factors might be associated with death. Further, we evaluated the impact of comorbidity, the workload of nurses, and the disease severity during the first 24 h of ICU admission by using the Charlson’s comorbidity index (CCI), the therapeutic intervention scoring system (TISS) 28, and the Simplified Acute Physiology Score (SAPS) II, respectively.

## Methods

### Study Design

This study was designed as a retrospective cohort study and conducted at a nine-bed neurological intensive care unit of a tertiary care university hospital. The local Ethics Committee evaluated the study protocol (Ethikkommission für das Bundesland Salzburg; 415-EP/73/534-2015). No patient consent was required due to the noninterventional design according to national regulations.

### Patients and Definitions

**Fig. 1:**
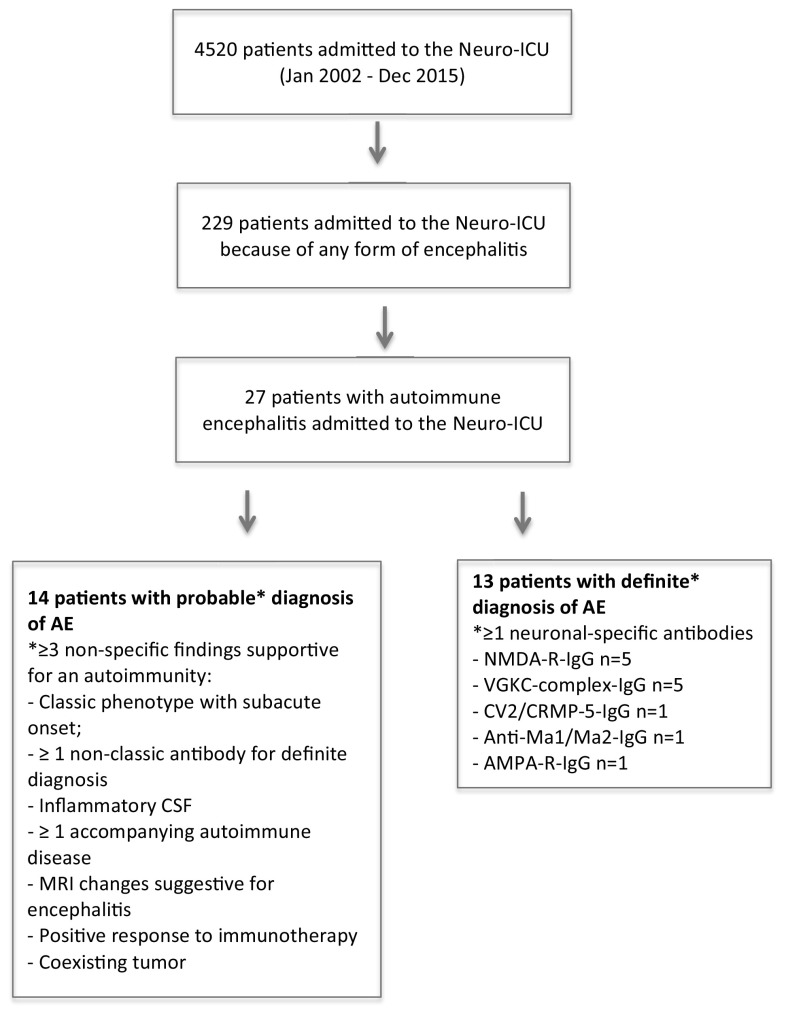
Patient selection flowchart. *ICU* intensive care unit, *AE* autoimmune encephalitis, *CSF* cerebrospinal fluid, *MRI* magnetic resonance imaging, *NMDA-R N*-methyl-d-aspartate receptor, *IgG* immunoglobulin G, *VGKC* voltage-gated potassium channel-complex, *CV*2*/CRMP5* collapsin response mediator protein 5, *AMPA-R* α-amino-3-hydroxy-5-methyl-4-isoxazolepropionic acid receptor

We reviewed all medical records of consecutive adult patients with encephalitis admitted to the ICU of whom the potential AE cases were re-diagnosed and categorized the patients as having either “definite” or “probable” AE based on adapted criteria suggested by Mittal and Graus [2, 11]. Patients in whom other acute neurological conditions were identified during the follow-up were excluded. Correspondingly, diagnostic criteria for the group “definite” were the detection of antibodies against the neuronal cell surface, synaptic, or onconeuronal protein in the cerebrospinal fluid (CSF) and/or serum. “Probable” encephalitis was diagnosed in patients, who did not fulfill the criteria for “definite” diagnosis but had at least three other supportive evidences for autoimmune CNS disease. Figure 1 provides an overview of the diagnostic criteria and the flowchart of patient selection.

### Data Collection

Comorbid conditions were quantified using the Charlson’s comorbidity index (CCI) [12]. Simplified Acute Physiology Score (SAPS) II was recorded as an indicator of disease severity during the first 24 h after ICU admission [13]. Therapeutic Intervention Scoring System (TISS) 28 was used to quantify the workload of nurses in the ICU [14]. “Definite” diagnosis of autoimmune encephalitis was based on the detection of antibodies against following antigens: Hu, Ma, Ri, Yo, Sox1, delta/notch-like epidermal growth factor-related receptor (DNER), collapsin response mediator protein 5 (CV2/CRMP5), glutamic acid decarboxylase (GAD65), dipeptidyl-peptidase-like protein-6 (DPPX), metabotropic glutamate receptor 1 (mGluR1), voltage-gated potassium channel-complex (VGKC) including leucine-rich glioma-inactivated 1 (LGI1), and contactin-associated protein-like 2 (CASPR2), *N*-methyl-d-aspartate (NMDA) receptor, γ-aminobutyric acid-B (GABA_B_) receptor, α-amino-3-hydroxy-5-methyl-4-isoxazolepropionic acid (AMPA) receptor, and amphyphysin. CSF was considered inflammatory if at least 2 of the following criteria were met: pleocytosis (≥5 white cells/ml), elevated IgG synthesis rate, increased protein concentration (≥70 mg/dl), and oligoclonal bands. Supportive MRI findings included mesial temporal or subcortical hyperintense changes on fluid-attenuated inversion recovery (FLAIR)/T2 imaging [1, 2]. We reviewed EEG reports and evaluated for the presence or absence of abnormal slow activities and epileptiform discharges. If the patient had more than one EEG, only the worst EEG was described. EEG evaluations refer to the new and validated criteria for status epilepticus [15, 16]. Patients were screened for malignancy using sonography and fluorodeoxyflucose positron emission tomography (FDG-PET). Mortality was determined according to ICU/hospital discharge or last follow-up patient records.

### Statistical Analysis

All statistical analyses were conducted using IBM SPSS version 21.0 (SPSS, Chicago, IL, USA). Descriptive methods were used to present data. Clinical, demographic, and mortality data between survivors and non-survivors (at last follow-up) were compared using the Fisher’s exact or Mann–Whitney *U* test, as appropriate. All reported *p* values were two-tailed and considered statistically significant at <0.05.

## Results

**Fig. 2:**
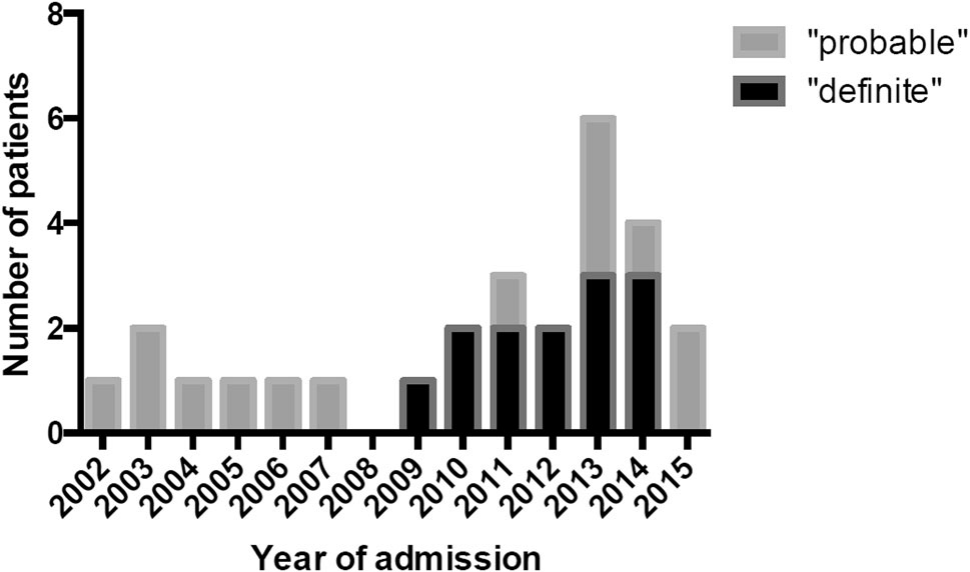
Temporal distribution of neurological intensive care unit (neuro-ICU) admissions in patients with autoimmune encephalitis. *Note* the columns represent absolute numbers, e.g., 3 patients in each subgroup for year 2013

From January 1, 2002 until December 31, 2015, 4520 patients were treated at the NICU of the study center. Of these 229 patients were admitted because some form of encephalitis. AE was diagnosed in 27 patients (0.6%), which comprise 67.5% of all AE patients diagnosed over the study period (including those not admitted to the ICU, *n* = 40 in total). The temporal distribution of NICU admissions of patients with AE is presented in Fig. 2.

### Clinical Presentations and Characteristics

A large proportion of AE patients had seizures prior to the NICU admission (*n* = 11, 40.7%). Other conditions preceding NICU admission were personality or behavioral changes (*n* = 7, 25.9%), subacute cognitive decline (*n* = 6, 22.2%), headache (*n* = 5, 18.5%), fever, and confusional state (*n* = 4, 14.8% each). Correspondingly, the most frequent diagnosis at the NICU among them was status epilepticus (*n* = 7, 26%), followed by delirium and respiratory failure (*n* = 4, 14.8% each), coma (*n* = 2, 7.4%), hemiparesis, and sepsis (*n* = 1, 3.7% each). One patient was admitted for the purpose of scheduled therapeutic plasma exchange. Demographic and clinical characteristics of critically ill patients are provided in Table 1. Further details of individual patients are shown in supplemental material for definite (e1) and probable (e2) autoimmune encephalitis.

**Table 1:** Demographic and clinical characteristics of critically ill patients with autoimmune encephalitis

Type of antibodies	Number (*n*)	Sex	Reason for ICU admission (*n*)	MRI changes (*n*)	Abnormal EEG (*n*)	Inflammatory CSF^a^ (*n*)	Malignancy (*n*)	Need for MV (*n*)	Death (*n*)
VGKCc	5	M4	SE = 4, RF = 1	LE = 3, *N* = 2	3	2	Pancreatic Ca = 1	1	1
NMDAR	5	F4	SE = 3, RF = Coma = 1	LE = 2, *N* = 3	4	0	Ovarian teratoma = 4	0	1
AMPAR	1	M	Delirium	LE	0	1	0	0	0
CV2/CRMP	1	M	Delirium	N	1	0	SCC lung = 1	1	0
Ma1/Ma2	1	M	SE	LA	1	0	Seminoma = 1	0	0
No antibody	14	M7	SE = 6, RF = Delirium = 2, Sepsis = Coma = Progressive ataxia = hemiparesis = 1	LE = 2, EL = 4, NSC = 4, LA = 2	9	9	Ovarian teratoma = 1, Lung CA = 1, Prostate Aca = 2, CRS = 1, CNS lymphoma = 1	4	3

*VGKC-c* voltage-gated potassium channel-complex antibody, *NMDA-R* *N*-methyl-d-aspartate receptor, *AMPA-R* α-amino-3-hydroxy-5-methyl-4-isoxazolepropionic acid receptor, *SE* status epilepticus, *RF* respiratory failure, *MRI* magnetic resonance imaging, *EEG* electroencephalography, *CSF* cerebrospinal fluid, *ICU* intensive care unit, *LE* limbic encephalitis, *EL* extra-limbic, *NSC* non-specific changes, *LA* limbic atrophy, *N* normal, *M* male, *F* female, *Ca* cancer, *Aca* adenocarcinoma, *SCC* small cell cancer, *CRC* colorectal cancer

^a^CSF was considered inflammatory if at least 2 of the following criteria were met

Pleocytosis (≥5 white cells/ml)

Elevated IgG index (>0.7)

Increased protein concentration (≥70 mg/dl)

Oligoclonal bands

### Associated Autoimmune Diseases and Tumors

Eight patients (29.6%) had coexisting systemic autoimmune disorders. Two patients (7.4%), who were seropositive for anti-NMDA-R antibody, had Hashimoto’s thyroiditis. The autoimmune disorders in patients with “probable” AE were the following: autoimmune thrombocytopenia, rheumatoid arthritis, systematic lupus erythematosus, vitiligo, Crohn’s disease, and dermatomyositis (*n* = 1, 3.7% each).

Thirteen out of 27 patients (51.8%) had the following underlying malignancies: ovarian teratoma (*n* = 5, 38.5%), prostate (*n* = 2, 15.4%), small cell and non-small cell lung cancer, pancreatic cancer, seminoma, CNS lymphoma, and rectal adenocarcinoma (*n* = 1 each, 7.7%). Four patients with ovarian teratoma were seropositive for anti-NMDA-R antibody, and the tumor was detected after AE diagnosis. The patient with seminoma was seropositive for anti-Ma1/Ma2 antibody, 1 out of 2 patients with lung carcinoma was seropositive for anti-CV2/CMPV-5 antibody. The remaining patients with tumors had other suitable criteria for AE, such as good response to immunotherapy, classical encephalitis with subacute phenotype, but without detection of specific antibodies.

One patient, who was seronegative for anti-neuronal antibodies, had first been admitted to the NICU because of limbic encephalitis in 2000, and was readmitted with hippocampal atrophy and rectal adenocarcinoma in 2013.

### Laboratory Findings

Cerebrospinal fluid was examined in all but one patient. This was the patient with a scheduled therapeutic plasma exchange.

In 13 patients (48.2%), the following anti-neuronal antibodies were detected either in serum, cerebrospinal fluid or both: anti-NMDA-R-IgG (*n* = 5), anti-VGKC-C-IgG (*n* = 5), anti-Ma1/Ma2-IgG, anti-CV2/CRPM-IgG, and anti-AMPA-IgG (*n* = 1 each).

Twelve patients (46.2%) had cerebrospinal fluid findings indicative of an inflammation (elevated IgG synthesis rate, high concentrations of total protein, pleocytosis, and oligoclonal bands), three of whom were categorized as having “definite” disease being additionally seropositive to VGKC-c (*n* = 2) and AMPA-R (*n* = 1) antibodies. An increased cerebrospinal fluid protein concentration (≥70 mg/dl) was detected in eleven (91.7%) of them (median value 133, range 78–290), pleocytosis (≥5 white cells/ml) in all twelve (median value 108.5, range 17–1048), elevated IgG synthesis rate in five (41.7%) (median value 12.6, range 6.18–22.5), and cerebrospinal fluid-specific oligoclonal bands in two patients.

The remaining six patients did not fulfill the criteria for inflammatory CSF but had one cerebrospinal fluid abnormality: one patient had an increased protein concentration (80 mg/dl), and five patients showed pleocytosis (median value 13, range 9–35). No cerebrospinal fluid abnormalities were found in eight (30.8%) patients.

### Brain Imaging

Specific T2-signal abnormalities (hyperintensities in affected brain regions, medial temporal lobes and/or subcortical regions) were detected in 15 patients (55.6%). These lesions were located in the limbic system in 11 patients (40.7%) and in extra-limbic regions in four patients (14.8%). Non-specific changes/leukoaraiosis were present in four (14.8%), and no abnormalities in eight patients (29.6%).

### EEG Findings

An EEG (prior to ICU admission) was performed in all but one patient “who was admitted for therapeutic plasma exchange.” Eighteen (69.2%) had at least one abnormality detected in the EEG. Seven patients (2 of which were seropositive to neuronal antibodies) had EEG pattern consistent with status epilepticus. The remaining seven patients (three with “definite” diagnosis) had abnormal slow activity (generalized *n* = 4, focal *n* = 3). Extreme delta brushes were found in the EEG of a patient with anti-NMDA receptor encephalitis.

### NICU Management, Secondary Complications

The median length of stay in the NICU was 5 days (range 1–85). Sixteen patients (55.6%) received immunotherapy. These treatments included corticosteroids (*n* = 7), intravenous immunoglobulin (*n* = 8), therapeutic plasma exchange (*n* = 12), and/or rituximab (*n* = 2). Eight improved on a combination treatment. Six patients required endotracheal intubation due to respiratory failure (*n* = 4, 66.7%), refractory status epilepticus, and/or palliative stenting for pancreatic cancer (*n* = 1, 16.7% each). The median duration of mechanical ventilation was 14 days (range 1–27). Percutaneous dilatational tracheostomy was performed in four (14.8%) patients, in three of them for a median duration of 12 days (range 1–19) following the endotracheal intubation, and immediately at the time of admission in one patient. The tracheostomy tube was removed after a median duration of 30.5 days (range 3–38). The summary of treatments is presented in Supplementary Material (Table_e-3).

The most common secondary complication was kidney failure (*n* = 4, 14.8%). Aspiration pneumonia occurred in 3 patients (11.1%). One patient developed chemotherapy-induced hepatopathy with a programmed cell death-1 (PD-1) inhibitor for the treatment of non-small cell lung cancer. Further complications included urinary infection and pneumothorax, respectively.

### Mortality and Causes of Death

**Fig. 3:**
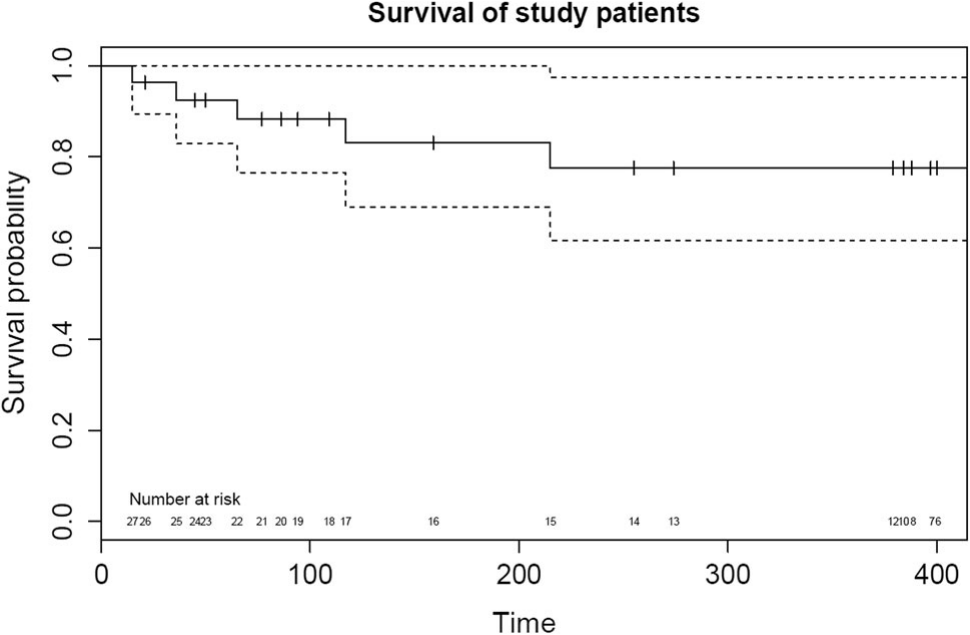
Survival curve of study patients. Survival curve (*solid line*) with 95% confidence intervals (*dotted line*) with the number at risk along the *x* axis

A comparison of survivors versus non-survivors is presented in Table 2. The mortality rate at last follow-up (median 1 year, range 1–15) was 18.5% (*n* = 5). One patient (20%) died because of multi-organ failure (sepsis) in the NICU, another two (40%) of sudden cardiac death, “one in the NICU and one after discharge in the hospital.” The two remaining patients died of cardiorespiratory failure and tumor progression after hospital discharge. The survival curve of study patients is presented in Fig. 3.

**Table 2:** Comparison of survivors versus non-survivors

Parameter	Total (*n* = 27)	Survivors (*n* = 22)	Non-survivors (*n* = 5)	*p* value
Age at admission (years)	55 (24)	72 (23)	50 (29)	0.006
Male gender [*n* (%)]	16 (59.3)	11 (50)	5 (100)	0.054
Charlson’s comorbidity index	3 (3)	2 (2)	8 (5.5)	0.001
Associated tumors [*n* (%)]	14 (51.8)	9 (40.9)	5 (100)	0.041
SAPS II	25 (22)	20 (18.3)	48 (30.5)	0.006
TISS-28	28 (6)	28 (6.5)	29 (7.5)	0.8
Noninvasive mechanical ventilation [*n* (%)]	6 (22.2)	2 (9.1)	4 (80)	0.004
Invasive mechanical ventilation [*n* (%)]	4 (14.8)	2 (91)	2 (40)	0.1
Plasma exchange [*n* (%)]	12 (44.4)	11 (50)	1 (20)	0.2
Vasopressor administration [*n* (%)]	17 (63)	15 (68.2)	2 (40)	0.2
Length of ICU stay (days)	5 (29)	5 (18.5)	15 (46)	0.04

All data are given as median values with interquartile range in parentheses, unless otherwise specified

*CCI* Charlson’s comorbidity index, *SAPS* simplified acute physiology score, *TISS* therapeutic intervention scoring system, *ICU* intensive care unit

## Discussion

We report the spectrum of clinical symptoms, underlying etiologies and mortality among critically ill patients with AE. Our study highlights the increasing number of ICU admissions due to AE over time. This observation is most likely the result of an increased recognition of this complex disease which eventually, can lead to the fact that the disease will cease to be rare [2]. Most importantly, we found that non-survivors had a longer ICU stay, higher need for mechanical ventilation, higher comorbidity and disease severity scores, and an increased rate of underlying malignancies than survivors.

To the best of our knowledge, only one study has so far reported on the ICU management of patients with definite and probable AE [11]. Compared to the latter cohort with 25 patients, our study population comprised elder patients and a higher percentage of male patients. Yet, the proportion of patients with detected anti-neuronal antibodies was similar. In accordance with the study from Mittal and colleagues, our study period covered a time when laboratory testing for many autoimmune antibodies were only scarcely available. This is likely to be the reason why the diagnosis of “definite” could only be established in about half of the cases in this cohort. Thus, the importance of the AE diagnosis based on clinical and paraclinical findings has to be appreciated, since early treatment measures are of critical importance [17].

In concert with the results from Mittal et al., we found seizures to be common (48 vs. 40.7%) clinical manifestation in AE patients requiring ICU admission. Behavioral changes and subacute cognitive decline, in contrast, occured predominantly among seropositive AE patients in our series (38.5 vs. 7.1%), but were more prevalent in the antibody-negative group of the mixed ICU cohort in Mittal’s study (38.5 vs. 66.7%, respectively). When interpreting these data, one has to take a referral bias innate to a NICU population into account.

The most common reason for ICU admission in our study population was status epilepticus (26%). Status epilepticus is a predictor of poor outcome in general for patients with encephalitis and specifically in critically ill patients with encephalitis [18, 19]. Remarkably, only one of the study patients admitted because of status epilepticus had lethal outcome. Interestingly, a recent French study reported that the occurrence of status epilepticus during the ICU stay in patients with anti-NMDA receptor encephalitis was not associated with poor outcome [20]. For GABA_A_ receptor antibody encephalitis high antibody titers were shown to be associated with a more severe clinical course and occurence of seizures, refractory status epilepticus, or both [21]. Thus, future studies should confirm and expand the relevance of antibody titers on the risk for the development of seizures.

We found that AE non-survivors (at ICU/hospital discharge/1 year) presented with more severe comorbidities, had more severe degree of critical illness as assessed by SAPS II scores, and had a higher requirement for mechanical ventilation than survivors. The latter finding is consistent with previous studies of critically ill patients suffering from encephalitis [18, 22–24]. Although not specific for an encephalitis population, comorbidity scores have been shown to be higher among neurological critical care non-survivors [25], albeit not in critically ill multiple sclerosis patients from our ICU, as we have studied previously [26].

As concerns the results of paraclinical investigations in our study population, only 46% showed abnormal cerebrospinal fluid findings. More than one-third of patients had MRI (FLAIR/T2) abnormalities supporting the diagnosis of AE. A recent multicenter study suggested that the cerebrospinal fluid analysis and MRI remains unremarkable in a significant proportion of patients with AE (37 and 41%, respectively) [27]. Together with these reports, our results emphasize the need for a multimodal approach and a high degree of clinical suspicion to diagnose AE. Therefore, improved recognition of the disease and awareness among medical practitioners is paramount.

As described in the literature, anti-NMDA receptor encephalitis typically affects female patients prior to the age of 30 years and is frequently accompanied by ovarian teratoma [28]. Accordingly, this was also the case in four of the five study patients diagnosed with anti-NMDA receptor encephalitis in our cohort. In all except one of these patients, teratoma was subsequently detected during the hospital stay. Notably, the only patient with anti-NMDA receptor encephalitis without cancer diagnosis died.

Although patients with encephalitis associated with cell surface antibodies have been shown to have a more favorable outcome compared to subjects with AE and intracellular antibodies [29], both deceased patients with “definite” autoimmune diagnosis in our series had anti-neuronal antibodies to cell surface. In both cases, death occurred only after hospital discharge.

Important limitations need to be considered when analyzing the results of our study. First, the retrospective study design provides lower diagnostic accuracy, and the relatively small sample size limits the generalizability of our findings. However, our results were comparable to the aforementioned series in most aspects [11]. Secondly, we acknowledge that the number of AE patients with specific neuronal antibodies might be underrepresented as a systematic retesting, and re-evaluation of both serum and CSF was inconsistent [30]. Moreover, patients with intracellular antigens may be low for the reason of management by oncologists. Finally, our study did not include patients younger than 18 years, while in one patient there were anamnestic hints pointing at earlier onset of AE. Therefore, we might have missed AE subtypes that more frequently occur in children. Concededly, studies with larger catchment area including other (medical, pediatric, etc.) ICU facilities would further complement our findings.

Our study suggests that clinical presentations and reasons for intensive care admission in critically ill AE patients are diverse, and the underlying conditions are dominated by status epilepticus. The association of comorbidity, malignancy, and the need for mechanical ventilation with adverse outcome should be considered in clinical practice and deserves further investigation in prospective studies. Further studies should also take the functional and cognitive status as outcome measure into account.

## Electronic supplementary material

Below is the link to the electronic supplementary material.

Supplementary material 1 (DOCX 21 kb)

Supplementary material 2 (DOCX 23 kb)

Supplementary material 3 (DOCX 25 kb)
