# Supplementary material for: Autoimmune Encephalitis at the Neurological Intensive Care Unit: Etiologies, Reasons for Admission and Survival
Source: Neurocrit Care. 2016 Dec 27;27(1):82–9. doi: 10.1007/s12028-016-0370-7 (PMC5524849; doi:10.1007/s12028-016-0370-7)
Supplement: Supplementary file 1 — Supplementary material 1 (DOCX 21 kb) [file 12028_2016_370_MOESM1_ESM.docx]

**\\]=-Table 2. Details of patients with „definite“ autoimmune encephalitis**

| #/Sex/Age, years | Reason for ICU admission | Symptoms prior to ICU admission | Ab | CCI | MRI changes | EEG changes | Inflammatory CSF* | Tumor | Respiratory support | PE/improvement | Length of ICU stay, days |
| --- | --- | --- | --- | --- | --- | --- | --- | --- | --- | --- | --- |
| 1/F/25 | SE | Confusion, behavioural and personality changes | NMDA-R | 2 | N | Slow activity | N | Ovarian teratoma |  | 13/N | 34 |
| 2/M/66 | SE | Seizures | VGKC-c/LGI1 | 2 | LE | N | N |  |  | 5/Y | 5 |
| 3/M/42 | SE | Seizures (focal) | Ma1/Ma2 | 2 | LA | Slow activity (temporal) | N | Seminoma |  | 4/N | 4 |
| 4/M/62 | Delirium | Confusion, subacute cognitive decline | AMPA | 10 | LE | N | Y |  |  | 6/N | 19 |
| 5/M/64 | SE | Personality and Behavioural charges, seizures | VGKC-c/LGI1 | 3 | LE | Slow activity | N |  |  | 5/Y | 5 |
| 6/M/66 | SE | Confusion | VGKC-c | 9 | N | EDs (fronto-parietal) | Y | Pancreas | MV |  | 11 |
| 7/M/87 | RF | Cognitive decline, ataxia | NMDA-R | 5 | LE | EDs | N |  |  | 7/N | 14 |
| 8/F/73 | RF | Cognitive decline, behavioural changes, seizures, headache | VGKC-c | 1 | N | N | N |  |  | 4/Y | 4 |
| 9/M/46 | SE | Seizures | VGKC-c | 2 | LE | Slow activity | Y |  |  |  | 5 |
| 10/M/64 | Delirium | Headache, confusion | CV2/CRMP-5 | 5 | N | Slow activity | N | Small cell lung cancer | MV |  | 85 |
| 11/F/29 | SE | Seizures, cognitive decline, behavioural changes | NMDA-R | 2 | LE | N | N | Ovarian teratoma |  | 10/Y | 38 |
| 12/F/30 | SE | Seizures, personality and behavioural charges | NMDA-R | 2 | N | Slow activity | N | Ovarian teratoma |  | 5/Y | 33 |
| 13/F/25 | Coma | Dizziness, headache, behavioural and cognitive changes | NMDA-R | 1 | N | Slow activity | N | Ovarian teratoma |  | 5/Y | 55 |

Notes: M, male; F, female; Y, yes; N, no; Ab, antibody; ICU, intensive care unit; SE, status epilepticus; RF, respiratory failure; LE, limbic encephalitis; EDs, epileptiform discharges; MRI, magnetic resonance imaging; EEG, electroencephalography; CCI, Charlson’s comorbidity index; MV, mechanical ventilation; PE, plasma exchange; CSF, cerebrospinal fluid.

* Inflammatory CSF was determined by the presence of >2 of the following:

- Protein ≥70 mg/dL
- IgG elevated rate > 8.18.1 mg/dL
- ≥5 white cells/ml
- oligoclonal bands
  ** Bolded rows represent non-survivors
