# Supplementary material for: Autoimmune Encephalitis at the Neurological Intensive Care Unit: Etiologies, Reasons for Admission and Survival
Source: Neurocrit Care. 2016 Dec 27;27(1):82–9. doi: 10.1007/s12028-016-0370-7 (PMC5524849; doi:10.1007/s12028-016-0370-7)
Supplement: Supplementary file 2 — Supplementary material 2 (DOCX 23 kb) [file 12028_2016_370_MOESM2_ESM.docx]

**Table 3. Details of patients with “probable” autoimmune encephalitis**

| #/Sex/Age, year | Reason for ICU admission | Symptoms prior to ICU admission | CCI | MRI changes | EEG changes | Inflammatory CSF* | Tumor | Respiratory support | PE (improve +) | Length of ICU stay | Fulfilled criteria, n |
| --- | --- | --- | --- | --- | --- | --- | --- | --- | --- | --- | --- |
| 1/F/37 | Hemiparesis | Headaches, speech impairment | 3 | ELE | N | Y | Ovarian teratoma | – |  | 5 | 3 |
| 2/F/61 | SE | Dizziness | 9 | NSC | N | N |  | – |  | 4 | 3 |
| 3/F/29 | SE | Seizures | 1 | LA | Slow activity (fronto-temporal) | N | Lung carcinoma | – |  | 1 | 4 |
| 4/M/58** | RF | Ataxia, nausea, speech impairment | 6 | ELE | N | N |  | MV |  | 3 | 3 |
| 5/M/83 | RF | Seizures | 13 | NSC | Slow activity (fronto-temporal) | N | CNS lymphoma | MV, PT |  | 36 | 3 |
| 6/F/53 | SE | Seizures, speech impairment, behavioural changes | 4 | LA | EDs | Y | Prostate adenocarcinoma | MV |  | 3 | 4 |
| 7/F/66 | Delirium | Fever | 3 | NSC | EDs | Y | Rectum adenocarcinoma | – |  | 11 | 3 |
| 8/M/72 | Coma | Headaches, fever, nausea | 8 | NSC | N | Y |  | MV, PT |  | 15 | 3 |
| 9/M/55 | Delirium | Dizziness, cognitive decline | 2 | LE | EDs | Y | Prostate adenocarcinoma | – | 5/+ | 5 | 5 |
| 10/M/47 | Progressive ataxia | Ataxia | 4 | ELE | §§ | Y |  | – | 6/+ | 4 | 3 |
| 11/M/55 | SE | Headache, fever | 1 | N | Slow activity (fronto-temporal) | Y |  | – |  | 9 | 3 |
| 12/M/55 | SE | Seizures | 2 | LE | EDS (parieto-temporal) | Y |  | – |  | 2 | 4 |
| 13/F/77 | Sepsis | Fever | 5 | ELE | Slow activity | Y |  | – |  | 3 | 3 |
| 14/F/42 | SE | Seizures | 4 | § | EDs | N | Ovarian teratoma | – |  | 5 | 3 |

M, male; F, female; Y, yes; N, no; ICU, intensive care unit; SE, status epilepticus; RF, respiratory failure; N, normal; LE, limbic encephalitis; LA, limbic atrophy; ELE, extra-limbic encephalitis; NSC, non-specific changes; EDs, epileptiform discharges; MRI, magnetic resonance imaging; EEG, electroencephalography; CCI, Charlson’s comorbidity index; MV, mechanical ventilation; PE, plasma exchange; CSF, cerebrospinal fluid.

* Inflammatory CSF was determined by the presence of >2 of the following:

- Protein ≥70 mg/dL
- IgG elevated rate > 8.1 mg/dL
- ≥5 white cells/ml
- oligoclonal bands

** Bolded rows represent non-survivors

§ MRI was not allowed in patient with implanted cardiac pacemaker.

§§ EEG was not performed
