# Supplementary material for: Autoimmune Encephalitis at the Neurological Intensive Care Unit: Etiologies, Reasons for Admission and Survival
Source: Neurocrit Care. 2016 Dec 27;27(1):82–9. doi: 10.1007/s12028-016-0370-7 (PMC5524849; doi:10.1007/s12028-016-0370-7)
Supplement: Supplementary file 3 — Supplementary material 3 (DOCX 25 kb) [file 12028_2016_370_MOESM3_ESM.docx]

Table_e-3. Summary of medical treatment

| # | - **AED** | - **Antiocoagulants** | - **Anti-hypertensives** | - **Vasopressors** | - **PPIs** | | - **Antibiotics (reason)** | - **Antipsychotics** | | - **Neuroleptics** | | - **Immunotherapy** | | | | | | | | | |  |
| --- | --- | --- | --- | --- | --- | --- | --- | --- | --- | --- | --- | --- | --- | --- | --- | --- | --- | --- | --- | --- | --- | --- |
|  |  |  |  |  |  |  |  |  |  |  |  | - **1st line** | | | | | | - **2nd line** | | | |  |
|  |  |  |  |  |  |  |  |  |  |  |  | - **PE** | | - **GCs** | | - **IVIG** | | - **CP** | | - **RTX** | |  |
| - 1 | - **+** | - **+** |  | - **+** | | - **+** | - **+** (Urinary infection) | | - **+** | | - **+** | | - **+** | | - **+** | | - **+** | | - **+** | | - **+** | |
| - 2 | - **+** |  |  | - **+** | |  |  | |  | |  | | - **+** | | - **+** | | - **+** | |  | |  | |
| - 3 | - **+** |  |  |  | |  |  | |  | |  | | - **+** | |  | |  | |  | | - **+** | |
| - 4 | - **+** | - **+** |  | - **+** | | - **+** |  | | - **+** | | - **+** | |  | |  | | - **+** | |  | |  | |
| - 5 | - **+** |  | - **+** | - **+** | |  |  | | - **+** | | - **+** | | - **+** | | - **+** | | - **+** | |  | |  | |
| - 6 | - **+** | - **+** | - **+** | - **+** | | - **+** |  | |  | |  | |  | | - **+** | |  | |  | |  | |
| - 7 | - **+** | - **+** | - **+** | - **+** | |  | - + (Pneumonia) | |  | | - **+** | | - **+** | | - **+** | | - **+** | |  | |  | |
| - 8 | - **+** | - **+** | - **+** | - **+** | | - **+** |  | |  | |  | | - **+** | | - **+** | | - **+** | |  | |  | |
| - 9 | - **+** |  | - **+** | - **+** | | - **+** | - **+** (Epididymitis) | | - **+** | |  | |  | |  | | - **+** | |  | |  | |
| - 10 | - **+** | - **+** |  | - **+** | | - **+** |  | | - **+** | | - **+** | | - **+** | | - **+** | |  | |  | |  | |
| - 11 | - **+** | - **+** |  | - **+** | |  |  | |  | |  | | - **+** | |  | | - **+** | |  | |  | |
| - 12 | - **+** | - **+** |  | - **+** | |  | - **+** (Colpitis) | | - **+** | |  | | - **+** | |  | |  | |  | |  | |
| - 13 | - **+** | - **+** |  | - **+** | |  | - + (Urinary infection) | | - **+** | | - **+** | | - **+** | |  | |  | |  | |  | |
| - 14 |  | - **+** |  |  | | - **+** | - + (Urinary infection) | |  | |  | |  | | - **+** | |  | |  | |  | |
| - 15 | - **+** | - **+** |  |  | | - **+** |  | |  | |  | |  | |  | |  | |  | |  | |
| - 16 | - **+** | - **+** |  |  | | - **+** |  | | - **+** | |  | |  | |  | | - **+** | |  | |  | |
| - 17 | - **+** | - **+** |  |  | | - **+** | - + (Pneumonia) | |  | |  | |  | |  | |  | |  | |  | |
| - 18 | - **+** | - **+** | - **+** |  | |  |  | |  | |  | |  | |  | |  | |  | |  | |
| - 19 | - **+** | - **+** |  |  | | - **+** |  | | - **+** | |  | |  | |  | |  | |  | |  | |
| - 20 | - **+** | - **+** | - **+** |  | | - **+** | - + (Urinary infection) | |  | |  | |  | | - **+** | |  | |  | |  | |
| - 21 |  | - **+** | - **+** |  | | - **+** |  | |  | |  | |  | |  | |  | |  | |  | |
| - 22 |  |  |  |  | |  |  | |  | |  | | - **+** | |  | | - **+** | |  | |  | |
| - 23 |  | - **+** | - **+** | - **+** | | - **+** |  | | - **+** | |  | | - **+** | | - **+** | |  | |  | |  | |
| - 24 | - **+** | - **+** |  | - **+** | | - **+** |  | |  | |  | |  | | - **+** | | - **+** | |  | |  | |
| - 25 | - **+** | - **+** |  | - **+** | |  |  | | - **+** | |  | |  | | - **+** | | - **+** | |  | |  | |
| - 26 |  | - **+** | - **+** | - **+** | | - **+** |  | | - **+** | |  | |  | | - **+** | |  | |  | |  | |
| - 27 | - **+** | - **+** |  | - **+** | |  |  | |  | |  | |  | | - **+** | |  | |  | |  | |

AED, antiepileptic drugs; PPI, protone pump inhibitor; PE, plasma exchange; IVIG, intravenous immunoglobulin G; GC, glucocorticoids; CP, cyclophospamide; RTX, rituximab.
